# Cesarean scar pregnancie

**DOI:** 10.11604/pamj.2018.30.228.14384

**Published:** 2018-07-26

**Authors:** Majdouline Boujoual, Driss Rahali Moussaoui

**Affiliations:** 1 Department of Obstetrics and Gynecology, Military Training Hospital Mohammed V, Rabat, Morocco

**Keywords:** Pregnancy, Cesarean scar, echography

## Image in medicine

A 35 years old woman presented at 8-week gestation for her first prenatal visit. She had no complaints at the time. While her past obstetrical history showed two prior cesarean sections. Transvaginal ultrasound was performed showing fortuitously a cesarean scar ectopic pregnancy with a gestational sac implanted in the myometrium between the bladder and the anterior wall of the uterus (A) and associated with a periovulatory hyper-vascularization in color doppler (B). This aspect was confirmed in 3D Ultrasound which showed a gestational sac bulging through the anterior wall of the uterus into the bladder with diminished myometrial layer between the bladder and the sac (C). The outcome was favorable after conservative surgical treatment by laparotomy associated with a triple preventive ligation because of the high risk of bleeding. Cesarean scar pregnancy is so a rare iatrogenic complication of cesarean section; however, her incidence is actually growing due to the increase in cesarean section rates. The diagnosis is usually made via ultrasound showing an empty uterine cavity and cervical canal, a gestational sac located anteriorly at the isthmus, and evidence of a functional trophoblastic/placental circulation on color Doppler. Early diagnosis is crucial to avoid severe vital and functional complications such as hemorrhage, early uterine rupture and hysterectomy, so its recognition is important to keep fertility of young woman.

**Figure 1:**
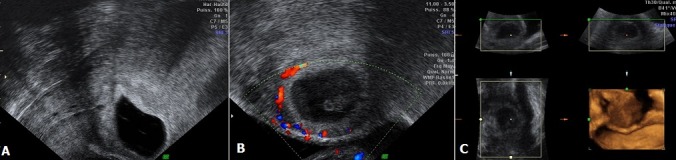
A) transvaginal echography in sagittal section showing a gestational sac implanted in the myometrium between the bladder and the anterior wall of the uterus with emptiness of the uterus and cervical canal; B) doppler ultrasound with color mode showing a periovulatory hyper-vascularization; C) ultrasound with 3D mode objectifying a gestational sac bulging through the anterior wall of the uterus into the bladder with diminished myometrial layer between the bladder and the sac

